# How do pregnant and lactating women, and young children, experience religious food restriction at the community level? A qualitative study of fasting traditions and feeding behaviors in four regions of Ethiopia

**DOI:** 10.1371/journal.pone.0208408

**Published:** 2018-12-05

**Authors:** Alessandra N. Bazzano, Kaitlin Storck Potts, Afework Mulugeta

**Affiliations:** 1 Department of Global Community Health and Behavioral Sciences, Tulane University School of Public Health and Tropical Medicine, New Orleans, LA, United States of America; 2 Taylor Center for Social Innovation and Design Thinking, Tulane University, New Orleans, LA, United States of America; 3 Department of Epidemiology, Tulane University School of Public Health and Tropical Medicine; New Orleans, LA, United States of America; 4 Department of Nutrition and Dietetics, Mekelle University, College of Health Sciences, Mekelle, Ethiopia; University of Ghana, GHANA

## Abstract

Maternal and child feeding behaviors are often rooted in family and sociocultural context, making these an important point of inquiry for improving nutrition and health over the life course. The present study explored the practice of fasting during religious periods in relation to eating patterns of pregnant and lactating women and young children in four regions of Ethiopia, a nation which has experienced rapid economic growth and marked improvement in health and nutrition outcomes over the last two decades. Qualitative data collection and analysis at community level illustrated conflicting areas of understanding and practice related to diets of children and pregnant and lactating women during fasting times, potentially leading to gaps in nutrition. Community participants described different understandings of fasting requirements for these vulnerable populations and associated social norms and doxa, not always in accordance with religious texts or published guidance. Useful behavior change strategies may be developed through these results to address the potential barriers to appropriate feeding patterns for pregnant and lactating women and young children in Ethiopia. This will include continuing to work with communities and religious leaders to clarify that religious doctrine promotes improved nutrition outcomes.

## Introduction

Ethiopia has made great strides in improving health and nutrition outcomes in recent years with a nearly 20-percentage-point decrease in stunting among 0–5 year olds from 2000 to 2016 [1]. However, the overall situation of child undernutrition in the country requires further reduction, with 38% of children under-5 stunted, 10% wasted and 24% underweight in 2016 [1]. Drought-related food emergencies have also highlighted the tenuous state of nutrition and food-security for vulnerable communities [2, 3].

Infant and young child feeding (IYCF) is closely linked with child nutritional outcomes and child development generally [4–7], and evidence-based child-feeding practices require scaling up throughout Ethiopia. In 2016, only 58% of children under-6 months were exclusively breastfed [1]. The percentage of children receiving a diet at the recommended level of diversity, defined as foods from four or more distinct groups in the previous 24 hours [8], remains a challenge for most families in Ethiopia, with only 14% children meeting this standard in 2016 [1].

Religious adherence is an important factor in health and nutrition. Qualitative research reveals that the health dictates of strict faiths are often negotiated [9]. Level of religiosity is also an important factor in how religious doctrine interacts with health or diet-related practices [10], wherein the study of strict adherents facilitates understanding of how doctrine may directly impact health outcomes [11].

The two most common religions in Ethiopia are the Ethiopian Orthodox Tewahedo Church (EOTC) and Islam with 43.5% and 33.9% adherents respectively in the population [12]. Both of these religions include fasting at prescribed times during the yearly calendar of religious events and holidays, and restrictions on food, as well as taboos related to consumption of animal source foods [13, 14]. However, the fasting traditions of these two religious groups are markedly different.

For adult members of the EOTC, there are approximately 250 fasting days in the year although not all are compulsory for everyone, with the average person fasting for around 180 days per year [14]. Most Wednesdays and Fridays are fasting days in addition to several periods of continuous fasting such as 55 days for Lent and a 43-day fast of the prophets. On fasting days in the EOTC all food and drink is abstained from until noon at the earliest when a small meal may be taken. Meat and animal products, including milk and eggs, are avoided entirely during fasting times in the EOTC. Church doctrine exempts children under-seven and pregnant women from fasting. There is extremely scant information related to EOTC fasting in relation to health, though some information is available on Eastern or Greek Orthodox fasting [15], which has similar tenets, though not the same schedule and fewer days of dietary or caloric restriction than EOTC fasting traditions.

By contrast, fasting in the Muslim tradition comprises one period throughout the year, the month long fast of Ramadan, when no food or drink is taken from sunrise to sunset [16]. A major distinction from that of EOTC fasting is that meats and animal source foods are allowed during the post-sunset evening meal of Iftar, which is meant to be substantial, festive, and shared with family and friends [17]. Exemption of children from fasting during Ramadan is the norm, but advice for pregnant and breastfeeding women is less clear and leaves the decision to the woman with the option to fast at a later time as an acceptable way to make up missed fasting [16]. Ramadan fasting is prohibited for menstruating women and translates to a prohibition for recently delivered women who are experiencing postpartum bleeding. Detailed material available through S1 File provides additional information on specific fasting doctrines. Personal level of religiosity and individual adherence to doctrine is an important factor which can influence actual practice of fasting [13].

Positive health-related markers have been found in relation to certain types of fasting among adults [18–22]. Fasting practices of adults who are parents may influence the amount and type of complementary foods that children are offered, particularly as a child transitions to family foods. The impact of fasting on health is not well described in the public health literature from low income countries in relation to child growth and anthropometry, though it has been studied in relation to many other topics related to adult health [15, 23–31].

Little research has focused on the impact of fasting on the diets of children in households where adults are fasting. The practice may influence breastfeeding, impacting the practice of exclusive breastfeeding of babies younger than 6 months or potentially altering the quantity or constituents of breastmilk received. Evidence on the impact of fasting on breastmilk composition and production is mixed [32, 33], and impacts will vary by fasting practices such as duration and types of dietary and caloric restriction.

If fasting requires dietary restriction of animal source foods, as in EOTC religious fasting, these foods may not be available in the household or the local community supply may decrease during periods of extended fasting (such as the 40+ day fasts), and therefore may not be available for child consumption. Animal source foods are an important source of macro- and micronutrients for children in low-income settings [34–36] and promoting provision of iron-rich animal source foods has been linked with improved dietary diversity and other important indicators of appropriate feeding [37].

The goal of this research was to explore fasting from the perspectives of mothers and other community members, and to utilize their descriptions of practices (and individual or social influences on those) from the study regions to understand and improve nutrition counseling and services. Given the complex nature of nutrition and fasting behaviors in relation to religious and sociocultural context, qualitative methods were chosen as the most appropriate to explore how participants perceive and understand these practices as part of their day to day lives [38].

## Materials and methods

### Sampling

A multi-method study of infant and young child feeding practices (IYCF) took place in selected four regions of Ethiopia from October through December 2015. The regions included Afar, Amhara, Benishangul-Gumuz, and Tigray. Geographic remoteness and resource limitations necessitated the survey include 2 zones per region. The zones were purposively selected based on IYCF factors to capture a range of experiences within region. The selected zones included Zones 1 and 4 in Afar, Eastern and North Western Zones in Tigray, South Wollo and West Gojjam in Amhara, and Assosa and Metekel in Benishangul-Gumuz. Within the selected zones, a household survey, cluster survey, food market survey, in-depth-interviews (IDI) and focus group discussions (FGD) were used to gather data on children less than 36 months of age and their mothers or caretakers living in rural areas. This paper utilizes results from the qualitative data collected during IDI and FGD.

### Data collection methods and tools

Qualitative data was collected from each cluster in the form of semi-structured IDIs and FGDs with health extension workers (HEWs) and caregivers. Interviews and focus group discussions were selected on the basis of being both the most appropriate for gathering qualitative data and the most feasible to undertake given the geographic distribution and distance [39]. The IDIs were conducted in person in the homes of the interviewed caregiver to facilitate opportunistic observations to corroborate findings. FGDs took place at common community meeting places. Having a child age 6–36 months living in the household was the inclusion criteria for caregiver participants. Participants were asked to refer to the youngest child when responding to study questions if multiple children met this age range. Caregivers and HEWs were selected for IDI and FGD participation based on maximum variation purposive sampling [40] in order to identify a range of participants to represent the array of experiences, characteristics, and perceptions that contribute to the behaviors under investigation. Characteristics for investigation included religion, parity, age, and livelihood. Identification of interview and FGD participants was aided by community leaders and could include those who also completed the quantitative survey. All caregiver IDI participants and most FGD participants were female. Some FGDs were conducted with male caregivers but these were not conducted in mixed company with female caregivers.

Semi-structured interview guides and FGD topic guides were developed in English, translated to Amharic, back translated to English, and pre-tested. Interviewers and FGD leaders all had previous experience conducting qualitative research and participated in a four-day training session prior to data collection. The training session covered protocol, interview and FGD techniques, and use of the guides. Conversations from FGDs and IDIs were audio recorded in the field and then transcribed by the interviewers and FGD leaders. The IDIs and FGDs from Benishangul-Gumuz and Amhara, and the FGDs in Tigray were transcribed verbatim.

The research team obtained approval from the Tulane University Health Sciences Institutional Review Board for secondary data analysis of data collected previously by a local non-governmental organization (NGO). The qualitative research staff of the NGO ensured that informed consent was obtained from all participants prior to data collection. Before commencing IDIs and FGDs, the interviewers and FGD leaders introduced themselves and indicated the study purpose. The interviewers/FGD leaders then read aloud an informed consent document. Interviewees were asked to sign the informed consent document prior to beginning data collection and verbal agreement was requested at FGDs. All data was de-identified upon importation for analysis to ensure confidentiality.

The data derive from 16 FGDs and 40 IDIs with caregivers and 32 IDIs with HEWs. These were performed across the eight zones sampled with 5 caregiver IDIs, 4 HEW IDIs, and 2 FGDs taking place in each zone by Ethiopian qualitative researchers who were fluent in local languages.

A naturalistic approach [41] to interpretive analysis guided this qualitative descriptive [42] exploration of data. Initially, a sample of transcripts was coded to develop a preliminary coding scheme for discussion, and this coding scheme was revised iteratively over the course of weekly discussions and memo-making by the analytic team of three experienced, female qualitative researchers. Content analysis was employed to understand experiences and behaviors related to optimal infant and young child feeding in the surveyed zones, and the sociocultural factors underlying these. Themes were derived from the data and not identified prior to analysis. NVivo software (Version 11) was used for analysis of the qualitative data. The COREQ guidance was utilized during to guide the presentation of this research [43].

## Results and discussion

### Description of participants

Female caregiver IDI participants were on average 29 years of age with fewer than two years of formal education. Half were Muslim, 48% were Orthodox and the remaining few were protestant. This ratio was similar among FGD participants. The mean age of the youngest child in the household of caregiver IDI participants was 18 months and this was slightly younger (17 months) among FGD participants. Of 127 FGD participants, 15% were male caregivers (from two FGDs conducted in Tigray). The majority of FGD participants were farmers (74%), while 21% were housewives. The 32 HEW IDI participants were 25 years old on average, all but two were female, 53% were Orthodox and 44% were Muslim. Additional descriptive characteristics of IDI and FGD participants are given in Table 1 below.

**Table 1:** Sociodemographic characteristics of the IDI and FGD participants from four regions of Ethiopia, 2018 (n = 199).

	Caregiver Interviews		HEW Interviews		Focus Group Discussions	
	% or mean (SD)	n	% or mean (SD)	n	% or mean (SD)	n
Percent female respondents	100.0	40	93.8	32	85.0	127
Religion of respondent						
Muslim	50.0	40	43.8	32	47.2	127
Orthodox	47.5		53.1		49.6	
Protestant	2.5		3.1		3.1	
Occupation of respondent						
Farmer	—	—	—	—	74.0	127
Housewife	—	—	—	—	20.5	
Merchant	—	—	—	—	5.5	
Average age of respondent in years	29.3 (4.9)	40	25.3 (4.1)	32	28.3 (8.1)	127
Average number of children of respondent	4.2 (1.7)	40	—	—	3.0 (2.1)	127
Average age of youngest child in months	18.0 (8.1)	40	—	—	16.7 (9.3)	126
Average number of years of education	1.6 (2.2)	40	—	—	—	—
Average time worked as a HEW in months	—	—	53.4 (39.0)	32	—	—
Average length of IDI/FGD in minutes	99.5 (46.6)	39	60.5 (15.8)	32	83.6 (12.2)	14
Total	100.0	40	100.0	32	100.0	127
	Caregiver Interviews		HEW Interviews		Focus Group Discussions	
	% or mean (SD)	n	% or mean (SD)	n	% or mean (SD)	n
Percent female respondents	100.0	40	93.8	32	85.0	127
Religion of respondent						
Muslim	50.0	40	43.8	32	47.2	127
Orthodox	47.5		53.1		49.6	
Protestant	2.5		3.1		3.1	
Occupation of respondent						
Farmer	—	—	—	—	74.0	127
Housewife	—	—	—	—	20.5	
Merchant	—	—	—	—	5.5	
Average age of respondent in years	29.3 (4.9)	40	25.3 (4.1)	32	28.3 (8.1)	127
Average number of children of respondent	4.2 (1.7)	40	—	—	3.0 (2.1)	127
Average age of youngest child in months	18.0 (8.1)	40	—	—	16.7 (9.3)	126
Average number of years of education	1.6 (2.2)	40	—	—	—	—
Average time worked as a HEW in months	—	—	53.4 (39.0)	32	—	—
Average length of IDI/FGD in minutes	99.5 (46.6)	39	60.5 (15.8)	32	83.6 (12.2)	14
Total	100.0	40	100.0	32	100.0	127

Throughout the data, participants expressed varied understanding of fasting requirements for different life stages and for each religion. Table 2 below indicates the range of practices illustrated by the data. Following the table, further detail is presented.

**Table 2:** Fasting practices reported by participants in 4 regions.

Population	EOTC	Islam
Menstruating women	Not reported	Exempt from fasting
Pregnant women	Fasting expected but not required by religious doctrine	Fasting expected but not required
Postpartum, breastfeeding women during first 5–10 days	-Fasting expected unless mother feels unable to support lactation -Fasting may take place at another time to compensate for missed period.	Exempt from fasting
Postpartum, breastfeeding woman after first 40 days	-Fasting expected -Fasting may take place at another time to compensate for missed period.	-Potentially exempt from fasting (if unable to support lactation) -Fasting may take place at another time to compensate for missed time
Newborn and infant	Fasting not expected	Fasting not expected
Children 1–3 years of age	-Fasting not expected but may be encouraged -Only fasting foods may be available in the home/community	Fasting not expected

### Fasting for pregnant and/or lactating women (PLW)

#### Social norms and views on fasting

Orthodox and Muslim participants alike reported that all adults are expected to fast during fasting times and that it is the norm for pregnant and breastfeeding women to fast, except for potentially a short period immediately postpartum.

Muslim participants described that pregnant women should fast and breastfeeding women are exempt from fasting for ten to forty days after birth.

*I’m expected to fast after one month and 10 days of delivery… In most cases*, *I fast the whole fasting periods allotted for a given year even though I’m breast feeding (IDI, Muslim Mother, age 30, Afar)*.

*The mother is expected to fast during her pregnancy and lactating periods but exempted from fasting the first forty days after delivery (IDI*, *Muslim Mother age 26, Afar)*.

*Pregnant women are supposed to fast and most mothers are fasting during their pregnancy (FGD*, *Muslim Mother, age 17, Afar)*.

A few participants indicated that fasting might impact perinatal health.

*If pregnant mother try to fast like this, it harms (FGD, Muslim Mother, age 19, Benishangul-Gumuz)*.

*For pregnant woman, when she is fasting, amount of blood decreases. The whole day she do not take water. Also, she do not take foods. When she take food/water after this delay, the pregnancy affected (FGD, Muslim Mother, age 25, Benishangul-Gumuz)*.

One participant contrasted with others on the requirements to fast, because of this issue:

*According to our religion, pregnant and lactating mothers are not expected to fast. Because during this time mothers are not only accountable for themselves but also for their children (FGD, Muslim Mother, age 37, Afar)*.

However, participants described that they felt an obligation to follow the fasting traditions of their religion, as another mother recalled.

*In this locality, mothers are expected to fast after 40 days of child birth. We give priority for the obligations and commitments expected from our religion. It is normal to see women who are fasting when pregnant (FGD, Muslim Mother, age 30, Afar)*.

#### Specific fasting practices

Orthodox participants also indicated pregnant and breastfeeding women are expected to fast except for approximately 7–12 days postpartum, when mothers are allowed to eat non-fasting (animal source) foods.

*Pregnant mothers are not exempt from fasting whereas lactating mothers can eat non fasting foods only up to 7 days after birth (IDI, Orthodox Mother, age 23, Tigray)*.

*It will be a sin if someone adult doesn’t fast… breastfeeding mothers are not required to fast until 12 days after giving birth. But pregnant women are required to fast (IDI, Orthodox Mother, age 29, Tigray)*.

*Pregnant women and women who are breastfeeding must fast… it is only allowed for ten days after giving birth to eat non-fasting foods (FGD, Orthodox Mother, age unknown, Amhara)*.

Some participants related the exemption from fasting immediately following birth to postpartum bleeding, similar to the fasting exemption for menstruating women involved with perceived impurity.

*During Ramadan, pregnant women are expected to fast during fasting days. Because they must fast and pray until they get birth. But they will be exempted from fasting during birth and 40 days right after birth. This is because according to our religion delivered women can’t be clean until 40 days since they flow a lot of blood (FGD, Muslim Mother, age unknown, Amhara)*.

*During Ramadan lactating women should fast during fasting days, because according to our religion women should be exempted if and only when they flow blood—during their menstrual cycle and 40 days right after their birth (FGD, Muslim Mother, age unknown, Amhara)*.

*If the women have bleeding (like menstruation), their fasting is not accepted (FGD, Muslim Mother, age unknown, Amhara)*.

*It is allowed for women to eat non-fasting food only for 40 days after they give birth until they will not have bleeding (FGD, Muslim Mother, age unknown, Amhara)*.

One mother from Afar mentioned that the post-partum exemption from fasting was related to physical recovery.

*A newly delivered mother and sick mothers are not expected to fast but any pregnant and lactating mother should fast. Immediately after delivery the mother is thought to be weak and depleted so that she will be exempted from fasting (FGD, Muslim Mother, age 35, Afar)*.

One mention was made on a modification to fasting for pregnant or breastfeeding mothers by not fasting the entire time during the day but abstaining from non-fasting foods.

*Though it is different among [other] lactating mothers, I eat fasting foods at times of fasting and never wait until 3:00PM. How I fast is abstaining from animal source foods (IDI, Orthodox Mother, age 32, Tigray)*.

Some mothers reported not fasting at all while breastfeeding infants. This participant from Afar explained that she did not fast for entire fasting periods while her child was young.

*I’ve had experience of breastfeeding my youngest baby during fasting. In most cases, I don’t fast the whole fasting periods allotted for a given year until the baby’s age turns two years. Since I’m expected to deliver enough amount of breastmilk for my child (IDI, Muslim Mother, age 27, Afar)*.

Several participants mentioned the need to compensate for missed fasting days by fasting at a later time.

*Pregnant and lactating women are expected to fast but if they can’t do that nothing will happen to them except priests will give them directions to fast on another time so as to be cleansed from sin (FGD, Orthodox Fathers, Tigray)*.

*No problem if she eats, she will compensate it some other time and no stigma or rejection happens to her (FGD, Muslim Mother, age 28, Afar)*.

*A mother who delivered recently does not fast and she will compensate and fast on another time (FGD, Muslim Mother, age 17, Afar)*.

*… there is no punishments for not fasting all seasons of fasting. But they are expected to replace (i.e. if she missed five regular fasting days she has to replace the missed number of days another time) when it is appropriate (when they are not breast feeding) for them (FGD, Muslim Mother, age 27, Afar)*.

This participant explained the different options for fasting, indicating pregnant and breastfeeding mothers in her community fast specifically by abstaining from animal source foods.

*There are two types of fasting. Fasting that staying without any foods and drinking till mid afternoon and eating enjera and fasting that avoids eating animal products. Here, lactating/pregnant mothers not use animal products and eat enjera. They do not use milk and meat (FGD, Orthodox Mother, age unknown, Benishalgul-Gumuz)*.

#### Rationale for fasting of PLW

Mothers of both religions reported perception of negative consequences if a person fails to fast. These were often related to individual or community understandings of religious doctrine.

*If someone from the family members fails to fast in fasting day, bad things will be happened to them. They will be punished by ‘akera’ [punishment after death] (IDI, Muslim Mother, age 27, Afar)*.

*In our religion, an adult person who fails to fast on a fasting day without any problem and illness will be asked by Allah and he will encounter a bad thing on earth (IDI, Muslim, Mother age 25, Benishangul-Gumuz)*.

*No, we do not eat on fasting day. We respect our religion. As our soul father told us from the books [holy book and other books used in the church], person who fail from fasting would face many problems. It is written in the books (FGD, Orthodox Mother, age unknown, Amhara)*.

*There is no question someone [must] fast but it is believed that if she declines to fast, she commits sin and becomes sinful (FGD, Orthodox Mother, age 29, Tigray)*.

One father also indicated spiritual rationale as the most important:

*Pregnant and lactating women also were expected to fast. … Nothing would not happen to this people apart from they should fast for their souls and spiritual benefits (FGD, Orthodox Father, age 38, Tigray)*.

Others mentioned possible stigmatization within the community and negative social consequences from family and neighbors for not fasting.

*If a pregnant/lactating woman does not fast, she would face shame from the community. She could not be able to go to church (FGD, Orthodox Mother, age unknown, Amhara)*.

*If a pregnant and lactating woman is not fasting during fasting days, they may face some social problems from their families or neighbors (FGD, Orthodox Mother, age unknown, Amhara)*.

*Since we all follow the same religion here, someone cannot deviate from what the religion requires… anyone not doing what is required won’t be accepted by the community (FGD, Orthodox Father, age 35, Tigray)*.

Participants also mentioned religious leaders as an influence on the rationale for fasting.

*If lactating or pregnant mother does not fast, the religious leaders pressure them and ask them to fast… (FGD, Muslim Mother, age 29, Afar)*.

*If [PLW] don’t fast… it is condemned by religious fathers. So it is impossible (FGD, Orthodox Mother, age 21, Benishangul-Gumuz)*.

*If we break fasting we regret and must get blessing from the spiritual fathers. Then we will be made to fast another time for the missed fasting (FGD, Orthodox Mother, age 22, Tigray)*.

*If they break fasting, [PLW] will report to their spiritual fathers and will be ordered to compensate after getting repentance or regret (FGD, Orthodox Father, age 43, Tigray)*.

Some mothers noted that fasting is related to their personal relationship with the divine.

*If pregnant and lactating mothers are not fasting during ‘Ramadan’, there are no consequences of this. People are fasting for the sake of making peace with Allah. Allah will pay back to their activities either in this world or in the eternal life (FGD, Muslim Mother age 35, Afar)*.

*Pregnant or lactating women would not face any problem from the society if she does not fast. Nobody from the society would ignore/stigma her. Only God would ask her (FGD, Muslim Mother, age unknown, Amhara)*.

One participant emphasized personal choice:

*If they are not fasting, nothing will happen by the community. But they may be told to fast. But if the lactating women don’t want to fast, the decision will up to her (FGD, Muslim Mother, age unknown, Amhara)*.

#### Perceptions of fasting in relation to lactation and breastfeeding

All participants agreed that infants and children were exempt from fasting in order to breastfeed during fasting days (as they normally would during non-fasting times).

*In our culture a newly delivered woman can be exempted from fasting only for ten days of after birth. After ten days she must fast. But she can breast feed her child (FGD, Orthodox Mother, age unknown, Amhara)*.

*In our culture pregnant and lactating women should fast on fasting days. They must fast at least until mid-day [instead of up to 3 pm]. But she must feed her child even if she is fasting (FGD, Orthodox Mother, age unknown, Amhara)*.

However, some reported difficulties breastfeeding while fasting. One participant described it this way:

*The child will breast feed during fast. **‘**Anaaf cimaadha’means breast feeding during fasting is difficult for me. How it is difficult is, I am fasting, [the baby] is breastfeeding. But I do not eat and drink all day. It is difficult. Difficult. Breast does not produce ‘hin mirgisuuf’ (IDI, Muslim Mother, age 34, Benishangul-Gumuz)*.

Other participants described difficulties:

*The baby is allowed to breastfeed during fasting days. But during the day time[mothers] are not eating, even not allowed to drink water but breastfeeding, and this is a difficult situation and [mothers] suffer a lot (IDI, Muslim Mother, age 26, Afar)*.

*We can breastfeed baby during fasting period but it affects health since we could not eat the whole day but feed breast to the baby (IDI, Muslim Mother, age 25, Afar)*.

Some participants explicitly described not having enough breast milk while fasting.

*During fasting days, I was breastfeeding my child. And it was one of my responsibilities and duties no matter how I felt hungry and uncomfortable… [Baby] was eating food items as much as she can… Because at that time I might not have enough breast milk to feed her since I was fasting (IDI, Muslim Mother, age 40, Amhara)*.

*It is so difficult to breastfeed while fasting for the reason that breast won’t produce enough milk and as a result [mother] feels pain as baby suckles and the baby cries a lot since he cannot get enough (IDI, Orthodox Mother, age 32, Tigray)*.

*On fasting and non-fasting days… the amount of breast milk become less in fasting times since [mothers] fast. We add more [solid] foods on fasting days to compensate that (IDI, Orthodox Mother, age 28, Tigray)*.

### Complementary feeding and fasting

Participants also explained that it is necessary to give additional supplements to babies who receive complementary food while fasting, due to perceived decrease in milk production.

*During fasting days we can breast feed our children … however, our breasts may not produce enough milk since we don’t feed well. So in order to supplement our children with food, we should prepare them some food items such as soup, macaroni, and pasta (FGD, Muslim Mother, age unknown, Amhara, South Wollo)*.

*The amount of breast milk may be reduced since [mother] fasts, but [they] can eat fasting foods like lettuce (IDI, Orthodox HEW, age 23, Tigray)*.

#### Religious doctrine for children

Participants generally reported that young children are excused from fasting rules, as exemplified by this mother from Afar:

*There is no problem of feeding a baby food like milk, cheese, butter, eggs, meat, during fasting time. No restriction on food type. (IDI, Muslim Mother, age 25, Afar)*.

The age when children are expected to begin fasting reportedly differed between the two religious groups, but participants unanimously stated that children less than 5 are excused. Most Muslim mothers noted 15 was about the age children begin fasting, though some mentioned age 8, and the Orthodox participants stated 7 to 10 years was the age children begin fasting.

*We are all Muslims and in our religion a child starts fasting when the child is between twelve and fifteen years depending on the parent’s preference and decision*. *No fasting before this age of the child (FGD, Muslim Mother, age 28, Afar)*

*According to the culture of Muslims children less than 15 years will be exempted from fasting since they are unable to fast the whole day as the adults. There are no obligations related to the religion which force them to fast (FGD, Muslim Mother, age 30, Afar)*.

*In our religion, Muslims must begin to fast when completed 7-years age and begin 8 years of age. Yes, [younger] children are exempt from fasting. Children less than eight years are exempted from fasting (FGD, Muslim Mother, age unknown, Amhara)*.

*Children are exempted from fasting during fasting days. They are not expected to fast until they get ten years old. They can even eat all non-fasting food items during fasting days (FGD, Muslim Mother, age unknown, Amhara)*.

#### Unintended consequences for children

Participants discussed the issue that if a mother herself is fasting, she would potentially refrain from preparing non-fasting (animal source) foods for her children, even if she believed they were not prohibited for the child, due to concern over contamination of the family’s utensils and dishes for those who are fasting.

*I am afraid that [cooking] utensils/materials may touch each other. I don’t give [child] butter, meat. I don’t eat it, so how do I give her? It is difficult. How do I prepare for them separately? The spoon, dish may touch each other for us. So [we are] frightened for contaminating the materials (IDI, Orthodox Mother, age 28, Benishangul-Gumuz)*.

One Health Extension Worker (HEW) from Amhara explained the concern mothers in her community have about preparing non-fasting foods.

*No, mostly mothers do not feed the same thing on fasting and non-fasting days. They have concern of contamination of their fasting food with the children non-fasting food. Mostly they prefer to give [animal] milk if they do have milk at home in the fasting days (IDI, Orthodox, HEW, age 28, Amhara)*.

Another HEW mentioned that mothers have asked them about how they should feed their children during fasting days, indicating it is a concern for some mothers.

*Some mothers ask us how to feed their children during fasting days. Because they don’t want to mix non fasting foods with that of fasting ones. Whenever mothers ask us such kind of question, we advise them to feed their children from separate pots to avoid confusion and fear of mixing non fasting foods with that of fasting ones (IDI, Orthodox, HEW, age 27, Amhara)*.

Some Orthodox mothers may feel comfortable preparing non-fasting foods for their children by using separate bowls and cooking utensils. This HEW mentioned these mothers share their methods with other mothers.

*Actually, some Christian mothers had the concern of contamination of fasting food with non-fasting food. But other Christians said we have separate cooking utensil for fasting and non-fasting food. They shared this experience for those mothers who had the concern of contamination (IDI, Muslim, HEW, age 24, Amhara)*.

One participant from Tigray mentioned mothers have concerns over preparing non-fasting foods for children on fasting days since they cannot taste the foods.

*The mother’s concern was how can she taste (for salt or flavor) a non-fasting/animal product while she is fasting and cooking or [worried for] the feeding tool to be mixed (IDI, Orthodox, HEW, age 23, Tigray)*.

The concept that women may not be allowed to prepare non-fasting foods for their children may come from others within the religious community. An Orthodox HEW from Tigray mentioned that these perceptions may be changing.

*People from the religious community (church) were say that a fasting person shouldn’t prepare a non-fasting food for anyone else. But this is changing and these church people are now teaching it is possible to wash hands with soap after preparing non fasting food (IDI, Orthodox, HEW, age 28, Tigray)*.

### Availability of animal source foods/nutrient dense foods during fasting

**Fig 1:**
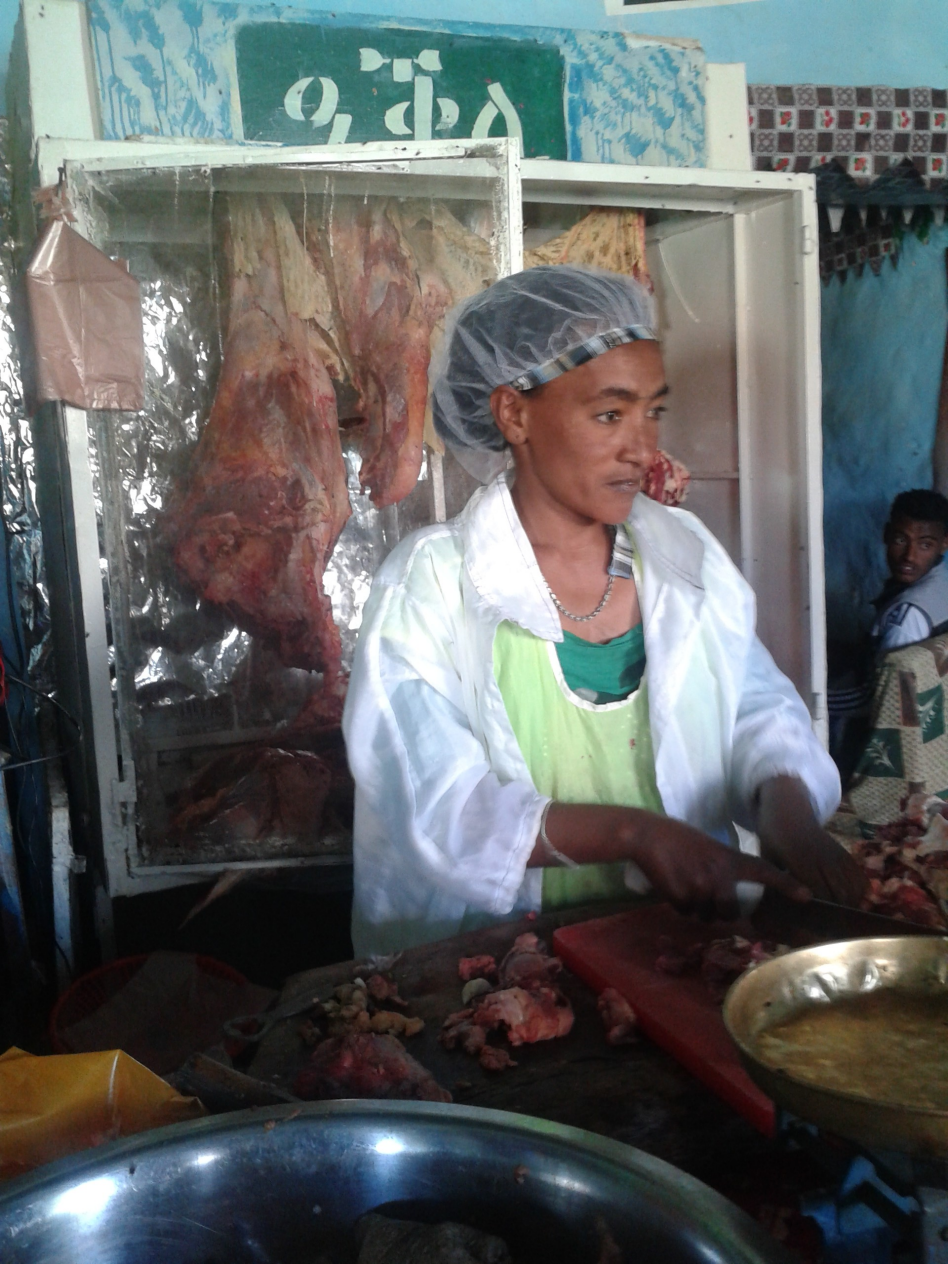
Butcher shop in study area selling animal source foods during non-fasting time*.*. The person in this photograph was not a study participant and has given permission for the image to be used widely and published.

According to participants, meat was reported to be scarcer and more expensive during fasting times, particularly during long periods of fasting like Lent in Orthodox communities, due to the lack of butchers willing to slaughter animals (fasting adults would be unlikely to do this). This decreased availability also increased the price of available meat. In Fig 1 below, a photo illustrates a butcher shop staffed by a woman in the study area (permission to be photographed was given, and the person in the photo was not a study participant) which is selling meat during non-fasting days.

Several participants confirmed the lack of availability of meat during fasting times:

*In the long fasting periods, meat is not available and he (youngest baby) does not eat meat (IDI, OrthodoxMother age 35, Amhara)*.

*Expenditure will be higher during non-fasting days because animal source foods are expensive (IDI, Orthodox, Mother, age 25, Tigray)*.

*Not all mothers give all the recommended food items especially meat due to shortages to fulfill that for children and problems of perception slaughtering/ buying meat exclusively for children in fasting seasons (IDI, Orthodox HEW age 28, Tigray)*.

*Church teachings don’t prohibit giving animal source foods (ASF) to children, except for meat, that meat will not be prepared for children during fasting [due to availability] (IDI, Orthodox, HEW, age 20, Tigray)*.

However, participants noted that eggs and milk may be more readily available ASF during fasting times in Orthodox communities.

*In the Christian families [they] can get especially egg and milk otherwise it is difficult to get meat because they do not slaughter animals during the fasting periods (IDI, Muslim, HEW, age 28, Amhara)*.

*There is no difference for children [diet during fasting] especially feeding milk and eggs remain allowed*. *But meat is not easily available in fasting days and is less probable to be fed to children. (IDI, Orthodox HEW age 28, Tigray)*

For Muslims, meat and animal source foods are allowed during the month of Ramadan and frequently eaten at the meal at the end of the fasting day. An HEW from Afar explained that diets may actually increase in variety during the month of Ramadan due to the celebratory nature of the evening meals.

*In Muslims religion the diet and variety during fasting times is better than the other days*. *Children under two years are not supposed to fast in the first place and during these fasting time more meat, soup, and other animal source foods will be available at home and the children will be fed during the fast times. The religious teaching has no influence on children feeding practice and no fasting for children under fifteen years. (IDI, Muslim HEW age 20, Afar)*

A Muslim mother indicates this phenomenon as well, by saying her food costs increase during Ramadan due to buying more things to eat than normal.

*During Ramadan our expense becomes higher than the normal circumstance. Because during which, we buy a lot of things to eat*. *(IDI, Muslim Mother age 40, Amhara)*

One HEW from Amhara noted the restrictive fasting diets of adults may benefit children’s diets due to lack of competition for meat in the household, and the decreased demand for meat in the community during fasting times.

*In fasting days, children of Christian family members may benefit a lot due to the fact that no one will [need to] share with children if they eat meat during fasting days*. *Animal source of food is available adequately because [it]can’t be used by adult during fasting days—especially in the Christian family members. (IDI, Muslim HEW age 29, Amhara)*

The data analysis identified diverse patterns of feeding for infants, young children and pregnant women. In addition, the participants expressed divergent understandings of sociocultural and theological norms around fasting rules for these groups. There are also distinctions between fasting traditions between the two main religious groups. In relation to EOTC fasting, one study is available on the impact on child nutrition, in which fasting was observed to be associated with decreased dietary diversity in one region [44]. However, there is little information in the peer-reviewed literature related to how fasting practices impact the diets of pregnant and lactating women or young children.

The effect of fasting on breastfeeding mothers is not fully understood. Some evidence of metabolic changes including increased metabolic stress in fasting breastfeeding mothers may place them at risk of fasting hypoglycemia [45, 46], potentially making it difficult for mothers to carry out daily activities including caring for and continuing to breastfeed their children. However, there is no direct evidence from human studies indicating that intermittent fasting results in decreased breast milk production. Observational studies on humans have given mixed results regarding changes in breastmilk composition from religious fasting of mothers [32, 47, 48].

Some animal studies have shown a potential decrease in milk production associated with decreased maternal energy intake, though there may be a minimum threshold at which this effect occurs [33]. More research is needed to understand the impact on milk production and composition of frequent intermittent fasting, which is the predominant pattern among the largest religious group in Ethiopia [14].

Our results indicate that some mothers felt their milk supply decreased during fasting. Mothers’ confidence about milk supply is closely associated with breastfeeding practices, so it is important that this does not negatively impact exclusive breastfeeding. Potentially using social and behavioral communication to support community members understanding of the mechanics of breastmilk production, especially the changes in volume and constitution to specifically meet the child’s unique need, could be important. Lactating women could also be encouraged to abstain from fasting, which is acceptable according to religious doctrine. In 2016, the EOTC developed a nutrition sermon guide in collaboration with USAID/ENGINE (Empowering New Generations to Improve Nutrition and Economic opportunities) which has been endorsed by the patriarch of the EOTC [49]. It encourages pregnant and lactating women and children under-seven years of age to eat nutritious foods, including animal source foods, during its official fasting periods. This is encouraging but its use and implementation of the actions it advises requires engagement with everyone in the community, especially religious leaders.

Fasting may inadvertently impact dietary diversity of young children, as some caregivers indicated they are not able to prepare ASFs for children on fasting days due to fear of contaminating family foods. A recent cross-sectional study found Ethiopian children of Orthodox Christian households during a fasting season whose mothers did not feed ASF to their child due to this fear, were 1.5 times less likely to have met the dietary diversity recommendation as compared to those who did not feed ASF for economic reasons [44]. In secondary analysis of data from the 2005 and 2011 Ethiopian DHS, Alive and Thrive found children from Orthodox families to be less likely to have consumed ASFs and meet the dietary diversity recommendations compared to children from other religions [50]. Furthermore, certain foods like meats, which are not allowed during fasting, become more expensive and more difficult to acquire during fasting times thereby limiting the ability to provide them to young children despite their exemption from fasting. One study found decreased calcium and vitamin B_2_, and decreased protein intakes in 6 to 36 month olds during an extended fasting time (Lent) compared to their intakes during non-fasting times [51].

There is limited research on the direct impact of fasting on nutritional markers in Ethiopian Orthodox children, and it may not necessarily exhibit detrimental impact. There is some evidence of improvement in health indicators of religiously fasting adults, mediated by intermittent vegetarianism or reduced consumption throughout the year [18, 52]. Rather than focusing on potential harms from religious fasting, this research highlights the importance of pinpointing the unique aspects of fasting for different groups that influence the diets of children.

While Ethiopia as a nation has made important population-level health and nutrition gains over the last decade, there may be social factors that could result in unaddressed disparities among vulnerable groups [53, 54]. Given the findings from this study, health and nutrition programming may helpfully be explored that addresses fasting practices in a supportive way for varied populations. Padela and colleagues present a framework for tailoring behavior change to religious affiliation [55]. Such an approach may be used in Ethiopia and other settings where religion is an important factor in social and behavioral context for health and nutrition. Table 3 below provides a summary of areas where formative research on nutrition and health interventions would be beneficial, based on the results of the study.

**Table 3:** Recommended domains of research for different fasting populations.

Population	Domains for formative research and intervention
Menstruating women	Promotion of adequate intake of important micronutrients
Pregnant women	Ensuring adequate intake of important micronutrients for non-fasting women, or micronutrient supplementation for women who fast during pregnancy
Postpartum, breastfeeding women during first 5–10 days	Ensuring adequate caloric and micronutrient intake for women during post partum recovery whether or not fasting
Postpartum, breastfeeding women 10–40 days and beyond	Ensuring adequate caloric and micronutrient intake for women during post partum recovery whether or not fasting during lactation
Newborn and infant	Supporting recommended breastfeeding and complimentary feeding practices, including timely initiation of breastfeeding, and exclusive breastfeeding; introduction of complementary foods including provision of adequate protein and micronutrients with or without animal source foods
Children 1–3 years of age	Supporting continued provision of sufficient and diverse diet, encouraging exemption from fasting for young children and provision of adequate protein and micronutrients with or without animal source foods

### Limitations

An important limitation of this paper is that participants were not asked to provide information on their personal level of practice of religion or strictness of adherence to the doctrines which may provide greater insight [56]; given the varied responses from participants, it is difficult to ascertain whether feeding behaviors are associated with adherence to religious guidelines and religiosity of individuals, or localized cultural or traditional practices. The qualitative portions of the study included caretakers of children ages 6 to 36 months, excluding children at younger ages. Although only participants with young children (under 36 months) were included, recall bias may result in inaccuracies in descriptions of past experiences. It is not expected that this would be differential by important participant characteristics. The study was conducted in purposively selected zones within 4 regions of the country, and practices may vary by region and zone based on factors that were not captured by this qualitative study.

## Conclusion

Fasting is an important practice for adherents to the two most common religions in Ethiopia and deeply rooted in sociocultural norms around feeding behaviors. Considering this alongside participants’ understandings and experiences may allow for useful behavior change strategies to be developed through formative research or human centered design to address potential barriers to recommended feeding patterns for pregnant and lactating women and young children.

## Supporting information

S1 File Additional detail on fasting protocols. (DOCX)
