# Supplementary material for: How do pregnant and lactating women, and young children, experience religious food restriction at the community level? A qualitative study of fasting traditions and feeding behaviors in four regions of Ethiopia
Source: PLoS One. 2018 Dec 5;13(12):e0208408. doi: 10.1371/journal.pone.0208408 (PMC6281271; doi:10.1371/journal.pone.0208408)
Supplement: S1 File — (DOCX) [file pone.0208408.s001.docx]

**Supplement 1.**

Fasting guidelines per religious affiliation

Islamic recommendations (per Reviewer) include:

- Pregnant women: Although pregnant women are encouraged to fast during Ramadan, it is not mandatory to fast during this period. Being in good health is a requirement for fasting during Ramadan, therefore if the health of a women or her unborn child appears to be threatened by fasting, she is exempt from fasting during that period. She is however required to make up for the lost fasting days as soon as she is able to do so (before the next Ramadan begins).
- Breastfeeding women: If a woman feels that she has the strength and can produce enough breastmilk for her child while fasting, then she is encouraged to fasting during Ramadan. However, a breastfeeding woman is also exempt from fasting but required to make up for the lost fasting days as soon as she is able to do so (before the next Ramadan begins).
- Children: Although children are encouraged to fast whenever they can (to cultivate the habit of fasting during Ramadan) they are not required to fast until they reach puberty. For girls, the age of their first menses is usually used to determine when they should begin (mandatory) fasting during Ramadan.

EOTC recommendations:

Addis Ababa, Ethiopia, January 12, 2016 *–* During the official annual meeting of the Holy Synod, the Patriarch of the Ethiopian Orthodox Tewahedo Church (EOTC), his Holiness Abune Matias I, formally blessed and endorsed a nutrition sermon guide which instructs to forgo fasting during the first 1000 days (from conception to age two) for mothers and their babies. The sermon guide, developed by EOTC in collaboration with USAID’s flagship multi-sector nutrition project, Empowering New Generations to Improve Nutrition and Economic opportunities (ENGINE), emphasizes the importance of consuming a nutritious diet including animal source foods in order to prevent maternal under nutrition and childhood stunting.

EOTC teachings have long prohibited pregnant and lactating women and children under seven from fasting, yet for many followers, this information is not widely known. The church has seven Canonical fasting seasons amounting to six and a half months per year when consumption of animal source foods is restricted. Communities rely on variable information and social norms which generally stress that fasting is required in order to adhere to ones beliefs.

To shed light on this issue and raise awareness on the effects of fasting practices on under-nutrition and childhood stunting, Ethiopian Orthodox Tewahedo Church Development and Inter-church Aid Commission (EOTC-DICAC) in collaboration with USAID/ENGINE held two consultative workshops with EOTC senior leadership and church scholars to clarify religious guidelines around fasting practices for children and pregnant and lactating women by developing a sermon guide based on church teachings, clearly outlining the laws and regulations related to nutrition and fasting. The sermon guide will be utilized to teach in dioceses, districts, parish churches, Sunday schools, theological colleges, clergy training centres and communities nationally.

The sermon guides highlights the following key points based on the four thematic focus areas:

1. **Pregnant women and nutrition during fasting seasons**

a. EOTC believes that pregnant women carry children in their womb during 275 days. During this period, they require nutritious foods, including animal source foods. Therefore, the Church allows pregnant women to eat nutritious foods, including animal source foods, from conception to birth. Priests/father confessors are responsible to teach and ensure that pregnant women and followers abide with these guidelines.

2. **Lactating mothers and nutrition during fasting seasons**

a. EOTC believes that lactating mothers should eat for two (for themselves and their baby). Therefore the Church allows lactating women to eat nutritious foods, including animal source foods, in order for their children to become healthy, as they are the future generation.

3. **Cooking Utensils and feeding practices for children under two**

a. As utensils used during fasting seasons can be washed and re-used, children under two years of age should not be forbidden from eating animal source foods, during fasting seasons.

b. The Church believes that for the health of children under seven, they should be exempted from fasting in her Orders, and therefore Priests are required to teach families (mothers, husbands and grandmothers) to feed children nutritious foods, including animal source foods and take care of them.

4. **Roles and Responsibilities of husbands, grandparents and religious fathers/priests**

a. As priests have the responsibility over their soul children, they should understand that pregnant, lactating mothers and children under two need special care during the first 1000 days and should teach them to feed children all types of nutritious foods, including animal source foods, without restrictions based on the eating Tradition of the Church.

b. Husbands and grandparents are the closest next-of-kin within the family unit, who should be taking care of their families (pregnant, lactating mothers and children under two).

c. They are required provide nutritious foods, including animal source foods and encourage them to prioritize eating these foods, during the first 1000 days. They should also share household chores and give all-round support, including taking care of their babies.

d. High priests and church scholars have the responsibility to give proper instructions to husbands and grandmothers, in this regard.

The sermon guide was distributed to the overall Holy Synod member Archbishops, who pledged their full support to ensure the sermon guide is utilized nationwide.

EOTC will conduct orientations on how to use the sermon guide to priests, preachers and deacons in her constituency, through the 53 dioceses’ General Managers, as a way to cascade the contents of the sermon guide to communities. This will ensure that the sermon guide principles, laws and regulations will be used consistently to minimize misperceptions among congregations and the public at large.

Kesis Samson Bekele (EOTC) – 0911639820

Email – [samson.bekele17@yahoo.com](mailto:samson.bekele17@yahoo.com)

Zelalem Mekuria (USAID-ENGINE) – 0911085613

Email – [zelalem.mekuria@savethechildren.org](mailto:zelalem.mekuria@savethechildren.org)
